# The Genetic Architecture of Hypertrophic Cardiomyopathy in Hungary: Analysis of 242 Patients with a Panel of 98 Genes

**DOI:** 10.3390/diagnostics12051132

**Published:** 2022-05-03

**Authors:** Róbert Sepp, Lidia Hategan, Beáta Csányi, János Borbás, Annamária Tringer, Eszter Dalma Pálinkás, Viktória Nagy, Hedvig Takács, Dóra Latinovics, Noémi Nyolczas, Attila Pálinkás, Réka Faludi, Miklós Rábai, Gábor Tamás Szabó, Dániel Czuriga, László Balogh, Róbert Halmosi, Attila Borbély, Tamás Habon, Zoltán Hegedűs, István Nagy

**Affiliations:** 1 Division of Non-Invasive Cardiology, Department of Internal Medicine, Faculty of Medicine, University of Szeged, Semmelweis u. 8, H-6725 Szeged, Hungary; lidiahategan@yahoo.com (L.H.); csabea88@gmail.com (B.C.); borbasjanos13@gmail.com (J.B.); tringer.anna@gmail.com (A.T.); palinkaseszti@hotmail.com (E.D.P.); viktoriadrnagy@gmail.com (V.N.); takacs.hedvig88@gmail.com (H.T.); 2 SeqOmics Biotechnology Ltd., Vállalkozók útja 7, H-6782 Mórahalom, Hungary; latinovicsd@seqomics.hu (D.L.); nagyi@seqomics.hu (I.N.); 3 Gottsegen National Cardiovascular Center, Haller u. 29, H-1096 Budapest, Hungary; nyolczasnoemi@gmail.com; 4 Military Hospital-State Health Center, Róbert Károly körút 44, H-1134 Budapest, Hungary; 5 Elisabeth Hospital, Dr. Imre József u. 9, H-6800 Hódmezővásárhely, Hungary; palinkasa@hotmail.com; 6 Heart Institute, Medical School, University of Pécs, Ifjúság útja 13, H-7624 Pécs, Hungary; faludi.reka@pte.hu; 7 Division of Cardiology, First Department of Medicine, Medical School, University of Pécs, Ifjúság útja 13, H-7624 Pécs, Hungary; rabai.miklos@pte.hu (M.R.); halmosi.robert@pte.hu (R.H.); habon.tamas@pte.hu (T.H.); 8 Division of Cardiology and Division of Clinical Physiology, Department of Cardiology, University of Debrecen, Móricz Zsigmond körút 22, H-4032 Debrecen, Hungary; nszgt@med.unideb.hu (G.T.S.); dczuriga@med.unideb.hu (D.C.); laszlobalogh76@yahoo.com (L.B.); borbelya@med.unideb.hu (A.B.); 9 Szentágothai Research Centre, University of Pécs, Ifjúság útja 20, H-7624 Pécs, Hungary; 10 Institute of Biophysics, Biological Research Centre, Eötvös Loránd Research Network, Temesvári krt. 62, H-6726 Szeged, Hungary; hegedus@brc.hu; 11 Department of Biochemistry and Medical Chemistry, Medical School, University of Pécs, Szigeti út 12, H-7624 Pécs, Hungary; 12 Institute of Biochemistry, Biological Research Center, Eötvös Loránd Research Network, Temesvári krt. 62, H-6726 Szeged, Hungary

**Keywords:** hypertrophic cardiomyopathy, genetic analysis, genetic variant

## Abstract

Hypertrophic cardiomyopathy (HCM) is a primary disease of the myocardium most commonly caused by mutations in sarcomeric genes. We aimed to perform a nationwide large-scale genetic analysis of a previously unreported, representative HCM cohort in Hungary. A total of 242 consecutive HCM index patients (127 men, 44 ± 11 years) were studied with next generation sequencing using a custom-designed gene-panel comprising 98 cardiomyopathy-related genes. A total of 90 patients (37%) carried pathogenic/likely pathogenic (P/LP) variants. The percentage of patients with P/LP variants in genes with definitive evidence for HCM association was 93%. Most of the patients with P/LP variants had mutations in *MYBPC3* (55 pts, 61%) and in *MYH7* (21 pts, 23%). Double P/LP variants were present in four patients (1.7%). P/LP variants in other genes could be detected in ≤3% of patients. Of the patients without P/LP variants, 46 patients (19%) carried a variant of unknown significance. Non-HCM P/LP variants were identified in six patients (2.5%), with two in *RAF1* (p.Leu633Val, p.Ser257Leu) and one in *DES* (p.Arg406Trp), *FHL1* (p.Glu96Ter), *TTN* (p.Lys23480fs), and in the mitochondrial genome (m.3243A>G). Frameshift, nonsense, and splice-variants made up 82% of all P/LP *MYBPC3* variants. In all the other genes, missense mutations were the dominant form of variants. The *MYBPC3* p.Gln1233Ter, the *MYBPC3* p.Pro955ArgfsTer95, and the *MYBPC3* p.Ser593ProfsTer11 variants were identified in 12, 7, and 13 patients, respectively. These three variants made up 36% of all patients with identified P/LP variants, raising the possibility of a possible founder effect for these mutations. Similar to other HCM populations, the *MYBPC3* and the *MYH7* genes seemed to be the most frequently affected genes in Hungarian HCM patients. The high prevalence of three *MYBPC3* mutations raises the possibility of a founder effect in our HCM cohort.

## 1. Introduction

Hypertrophic cardiomyopathy (HCM) is a common inherited cardiac disease, defined by the presence of left ventricular hypertrophy (LVH) in the absence of other causal cardiac or systemic conditions [1]. It is the most frequent inherited cardiac disorder, with prevalence estimated as being between 1:200 and 1:500 individuals [2,3]. On genetic grounds, HCM is an autosomal dominant Mendelian disease, characterized by variable expressivity and penetrance. The first HCM-causing gene, the beta myosin heavy chain gene (*MYH7*), was identified in 1989 [4], and a further seven causative sarcomeric genes (*MYBPC3*, *TNNT2*, *TPM1*, *MYL2*, *MYL3*, *TNNI3*, and *ACTC1*) [5,6,7,8,9,10] were detected throughout the 1990s. Since then, more than 1000 variants in these eight sarcomeric genes have been linked to HCM.

Over the last 20 years, candidate gene research studies assessing genes with a hypothetical role in HCM implicated over 40 additional, mainly non-sarcomeric genes in HCM. This process has been accelerated by the advent of novel DNA sequencing technologies (next generation sequencing, NGS), which revolutionized the field of genomics by allowing accurate, rapid, and high-throughput screening but, in turn, generated tremendous amounts of variants of unknown significance (VUS), which were difficult to interpret because of insufficient data. However, genetic diagnosis is important for the clinical management of HCM patients and their families, primarily as it facilitates the identification of mutation carriers in the families. As a positive genetic finding confirms the etiology of the disease and enables mutation-specific family screening, it is recommended by clinical guidelines [1,11].

The majority of data on the genetic background and disease gene distribution in HCM patients come from large referral centers from Western Europe and the U.S., and data are scarce on Central-European populations. Reports from the region assessing small cohorts of HCM patients have been published from Slovakia (8 pts) [12], Romania (54 pts) [13], and Poland (29 pts) [14] and a larger cohort from the Czech Republic (336 pts) [15].

Understanding of the genetic architecture of HCM is of utmost importance in every geographical region as the genetic background of HCM may be variable across different populations. Hungarians possess a unique genetic constellation as they have ancient roots originating from the Volga-Ural/West Siberian region. Although the present-day Hungarian gene pool is highly similar to that of the surrounding European populations, a limited portion of specific Y-chromosomal lineages link modern Hungarians with populations living close to the Ural Mountain range on the border of Europe and Asia [16]. Therefore, we aimed to perform a nationwide large-scale genetic analysis of a previously unreported Hungarian HCM cohort using targeted resequencing of a comprehensive cardiomyopathy gene panel comprising 98 genes.

## 2. Patients and Methods

### 2.1. Patients and Clinical Evaluation

The study cohort comprised unrelated, consecutively evaluated patients with HCM referred to collaborating cardiovascular centers, establishing a nationwide framework, including University of Szeged, University of Pécs, University of Debrecen, and the Military Hospital-State Health Center, Budapest.

Patients underwent 12-lead ECG, echocardiography; ambulatory ECG monitoring as baseline assessment; and further specialized cardiology examinations when indicated. HCM was diagnosed in probands when the maximum left ventricular wall thickness (MLVWT) measured by two-dimensional echocardiography was 15 mm or more in at least one myocardial segment, in the absence of other diseases that could explain LV hypertrophy. Blood samples were collected at routine clinic visits, and DNA was isolated from peripheral blood lymphocytes.

### 2.2. Gene Panel Selection

Samples were analyzed with a custom-made cardiomyopathy gene panel, including the following sets of genes, categorized as reported previously [17].

- genes having definitive (8 genes: *MYBPC3*, *MYH7*, *TNNT2*, *TNNI3*, *TPM1*, *ACTC1*, *MYL2*, *MYL3*) or moderate (3 genes: *CSRP3*, *TNNC1*, *JPH2*) evidence for HCM association (HCM genes);
- intrinsic cardiomyopathy genes (2 genes: *ACTN2*, *PLN*);
- syndromic genes, where isolated left ventricular hypertrophy (LVH) may be seen (7 genes: *DES*, *FHL1*, *RAF1*, *PRKAG2*, *LAMP2*, *PTPN11*, *TTR*);
- genes having definitive (10 genes: *BAG3*, *LMNA*, *RBM20*, *TTN*, *DSP*, *PKP2*, *DSG2*, *DSC2*, *JUP*, *TMEM43*) or moderate (2 genes: *NEXN*, *VCL*) evidence for DCM and/or ARVC association (DCM/ARVC genes);
- other genes, with limited but reported evidence for disease association with cardiomyopathies or candidate genes.

Altogether, 98 genes were analyzed (Supplementary Table S1). Variants in non-HCM genes were reported only in case of pathogenic (P) or likely pathogenic (LP) variants.

### 2.3. Sequencing

Coding sequences and exon–intron boundaries of all included genes were determined by next-generation sequencing using Agilent’s SureSelect technology with custom-made 120-mer RNA baits (designed using SureDesign), specific to the target region (Agilent Technologies, Santa Clara, CA, USA). Briefly, DNA was fragmented by the Covaris S2 System (Covaris Inc., Woburn, MA, USA), and the libraries were prepared using a SureSelect XT Reagent Kit (Agilent Technologies, Santa Clara, CA, USA), according to manufacturer’s instructions. All the purification steps were performed using AmPureXP Beads (Beckman Coulter Inc., Brea, CA, USA); all quality measurements were performed on a TapeStation 2200 instrument (Agilent Technologies, Santa Clara, CA, USA) and aa Qubit (Thermo Fisher Scientific, Grand Island, NY, USA). The concentration of each library was determined using the KAPA Library Quantification Kit for Illumina (KAPA Biosystems Inc., Wilmington, MA, USA). Sequencing was performed on Illumina HiSeq or NextSeq 500/550 instruments (Illumina Inc., San Diego, CA, USA) using single-end reads (1 × 150 bp), generating ~3 million reads for each sample. Variants, identified by targeted resequencing, were validated by standard capillary sequencing using custom-designed primers. Briefly, amplicons were generated using Platinum SuperFi polymerase and were subsequently sequenced using the BigDye Terminator v3.1 Cycle Sequencing Kit on the 3500 Genetic Analyzer platform (all from Thermo Fisher Scientific, Grand Island, NY, USA), following manufacturer’s instructions.

### 2.4. Bioinformatic Analysis

Mapping of the 150 bp Illumina reads were accomplished by Genomic Workbench ver. 11 (CLC Bio, now part of Qiagen), using the human genome assembly GRCh38 as reference sequence. Variant calling and variant annotation were performed by the same software. The functional impact of amino acid changes caused by missense mutations was predicted by SIFT and PROVEAN programs. Nucleotide and amino acid changes were reported according to the Ensembl database.

### 2.5. Interpretation of Variants

Identified variants were evaluated according to the standards for the interpretation of sequence variants issued by the American College of Medical Genetics and Genomics and the Association for Molecular Pathology (ACMG/AMP) in 2015 [18], with published gene-specific adaptation [19], and they were classified as benign (B), likely benign (LB), a variant of unknown significance (VUS), likely pathogenic (LP), and pathogenic (P). Variants were interpreted using CardioClassifier [20], an automated and interactive web tool that supports disease-specific interpretation of genetic variants in genes associated with inherited cardiac conditions and assessing ClinVar variants entries (https://www.ncbi.nlm.nih.gov/clinvar/, accessed on 15 January 2022). A score for every ClinVar entry was assigned (benign: 1, likely benign: 2, variant of unknown significance: 3, likely pathogenic: 4, and pathogenic: 5), and the average of the scores was calculated. Variants with an average score of >4.5 were classified as P variants, and variants with an average score between 3.5 and 4.5, between 2.5 and 3.5, between 1.5 and 2.5, and <1.5 were classified as LP, VUS, LB and B variants, respectively. In case of discrepancy between CardioClassifier and ClinVar interpretations, a final verdict was reached by assessing clinical evidence for disease causation (especially data on the number of affected individuals with the condition and evidence for co-segregation). In case of novel variants with no ClinVar entry and not covered by CardioClassifier, the Varsome (https://varsome.com/, accessed on 15 January 2022) on-line interpretation program was used.

## 3. Results

### 3.1. Study Population

242 unrelated index patients (127 (52%) men) with a clinical diagnosis of HCM were studied. At the time of diagnosis, the mean age was 44 ± 11 years, and the mean maximal left ventricular wall thickness was 23 ± 7 mm. The main demographic and clinical characteristics of the patients are summarized in Table 1.

### 3.2. Summary of Sequence Data

The median value of the per-sample average read depth (number of reads mapped on a 100-bp target region) across the samples was 392. Only 6 out of 242 samples had an average read depth lower than 50, with a minimum of 13.96% of the overall sequenced target regions covered to a depth of 40 or more and 98% to a depth of 20 or more.

### 3.3. Patient-Level Variant Analysis

The patient-level variant analysis (one variant per patient) is reported in Table 2.

#### 3.3.1. Patient-Level Variant Associations in HCM Genes

Out of the 242 study patients, 90 (37%) carried P/LP variants. The percentage of patients with P/LP variants in genes with definitive evidence for HCM association was 93%. Most of the patients with P/LP variants had a mutation in *MYBPC3* (55 pts, 61%), followed by patients with P/LP variants in *MYH7* (21 pts, 23%). P/LP variants in other genes were found in less than 3% of the patients.

Double mutations with P/LP were present in four patients (1.7%): three patients with two LP/P variants in *MYBPC3* (composite heterozygotes) and one patient with simultaneous P/LP variants in *MYH7* and *MYBPC3* (double heterozygote).

Of the patients without P/LP variants, 46 (19%) carried a VUS. The majority of these patients had a VUS in *MYBPC3* (9 pts, 20%) and in *MYH7* (11 pts, 24%). Patients with a VUS in other genes with >5% were patients with a VUS in *TPM1* (4 pts, 9%), *PRKAG2* (4 pts, 9%), *MYL2* (3 pts, 7%), *MYL3* (3 pts, 7%), and *CSRP3* (3 pts, 7%). The percentage of patients with VUS variants in genes with definitive evidence for an HCM association were 76%.

#### 3.3.2. Patient-Level Variant Associations in Syndromic Genes with Isolated LVH, DCM/ARVC Genes, and Other Genes

Six patients carried non-HCM P/LP variants, with two in *RAF1* (p.Leu633Val, p.Ser257Leu), one in *DES* (p.Arg406Trp), one in *FHL1* (p.Glu96Ter), one in *TTN* (p.Lys23480fs), and one in the mitochondrial genome (m.3243A>G).

**Figure 1:**
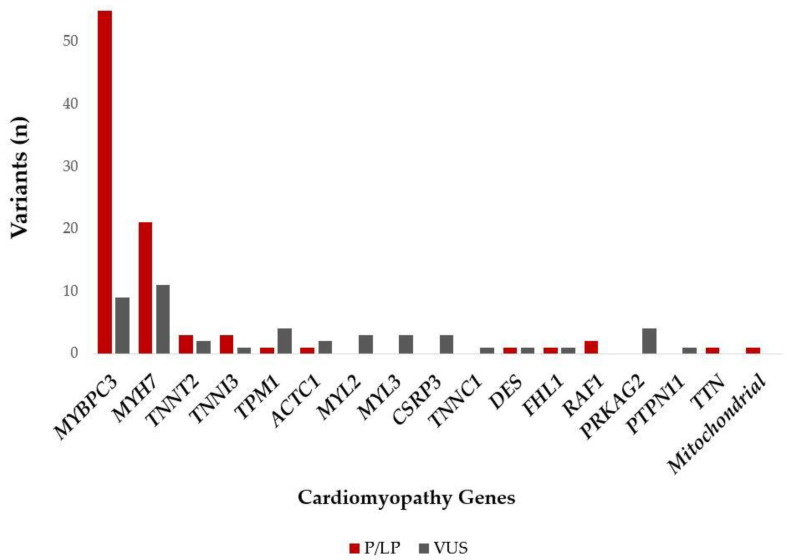
P/LP and VUS (in patients without P/LP variants) variants identified in the different cardiomyopathy genes among the Hungarian HCM patients. Abbreviations: P/LP: pathogenic/likely pathogenic variant; VUS: variant of unknown significance.

Two pathogenic variants (*ACADVL* p.Arg615Gln, *TSFM* p.Gln286Ter) were found in a heterozygous state and as a second variant in addition to another P/LP *MYBPC3* variant and, therefore, were not considered as disease-causing. The *SDHA* p.Met1? variant was identified in two patients, with one of them in a homozygous and the other one in heterozygous state. Since this alteration had been reported in an individual with Leigh syndrome [21], as well as in individuals with paraganglioma and gastrointestinal stromal tumor (GIST), the importance of this variant in relation to HCM is unclear. The patient-level variant analysis is summarized in Figure 1.

### 3.4. Gene-Level Variant Analysis

The gene-level variant analysis (all variants included only once if present in more patients) is reported in Table 3.

Altogether, 130 distinctive variants were detected in the patients (Supplementary Table S2). Out of the 130 variants, 52 (40%) were P/LP variants, while 61 (47%) and 17 (13%) were VUS or B/LB variants, respectively. If all the variants, including multiple-occurring variants, are considered, a total of 230 variants were identified in 159 patients (1.4 variants/patient).

The most distinctive variants were identified in *MYBPC3*. Out of the 37 variants, 59% was a P/LP variant, and 24% was a VUS. Out of the 30 distinct *MYH7* variants 57% was a P/LP variant, while 40% was a VUS. In all the other HCM genes, except *TNNI3*, VUSs were more prevalent than P/LP variants. This was also true for the syndromic genes, with isolated LVH.

A total of 82% of all P/LP *MYBPC* variants were frameshift, nonsense, and splice-variants. In all the other genes, missense mutations were the dominant form of variants (Supplementary Table S3).

A total of 31 novel variants were identified; six of them were P/LP variants, with four in *MYBPC3*, one in *FHL1,* and one in *TTN* (Supplementary Table S3).

### 3.5. Possible Founder Mutations in the Patient Cohort

There were three P/LP variants identified in more than three patients. These included the *MYBPC3* p.Gln1233Ter in 12, the *MYBPC3* p.Pro955ArgfsTer95 in 7, and the *MYBPC3* p.Ser593ProfsTer11 in 13 patients, comprising 36% of all patients with identified P/LP variants. These patients were seemingly unrelated, raising the possibility of a possible founder effect for these mutations.

## 4. Discussion

In our work, we reported the results of the genetic analysis of a previously not published Hungarian patient cohort with HCM. The detection rate of P/LP variants in our patient cohort was 37%, with an additional 19% of patients carrying a VUS, among patients who had no P/LP variants. This figure for the detection rate is similar to literature data, with detection rates in the range of 21–43% having been reported (in cohorts with more than 100 screened patients) [13,15,22,23,24,25,26,27]. The two major HCM genes in our cohort proved to be *MYBPC3* and *MYH7* genes, as the majority of the patients in our cohort carried P/LP variants in *MYBPC3* (61%), followed by patients with P/LP variants in *MYH7* (23%). This finding is also similar to other reported HCM disease-gene distributions, with *MYBPC3* and *MYH7* genes being the most frequently affected disease-associated genes [13,15,22,23,24,25,26,27]. The percentage of patients with P/LP variants in sarcomere genes with definitive evidence for HCM association was 93% in our cohort, which is also similar to reported data. The variant-level analysis indicated that frameshift, nonsense, and splice-variants made up 82% of all P/LP *MYBPC3* variants, while in all the other genes, missense mutations were the dominant form of variants. This observation is also very well in line with published data.

The results of our study also support the observation that sequencing expanded panels with an increasing number of genes offers limited additional sensitivity. Beyond genes with definitive or moderate evidence for HCM association and syndromic genes with isolated LVH, we identified only one patient with a P variant in the *TTN* gene and another one with a mitochondrial mutation. Data from the Laboratory for Molecular Medicine (Partners Healthcare Personalized Medicine, Boston, MA, USA) indicated that an expanded gene panel encompassing more than 50 genes identified only a very small number of additional pathogenic variants beyond those identifiable in their original panels, which examined 11 genes with a detection rate of ~32% among unselected probands [22]. More importantly, Walsh et al. sequenced 31 genes implicated in HCM in a large prospective HCM cohort (n = 804) and found no significant excess of rare (minor allele frequency <1:10,000 in ExAC) protein-altering variants over controls for most genes tested and concluded that novel variants in these genes are rarely interpretable. Indeed, extended gene panels rarely identify P/LP variants outside of the core-genes, which can be interpreted with a high degree of certainty [28].

An interesting finding of our study is that we identified three P/LP variants in *MYBPC3*: *MYBPC3* p.Gln1233Ter, p.Pro955ArgfsTer95, and p.Ser593ProfsTer11, which made up 36% of all patients with identified P/LP variants. These patients were seemingly unrelated, which raised the possibility of a putative founder effect for these mutations. Although these variants have been reported worldwide [29,30], they were identified mostly in isolation, without an established founder effect, except for the p.Pro955ArgfsTer95 mutations, which have been observed in 1.6% of the Dutch HCM population. As described in Dutch [31] and Finnish [32] populations, the *MYBPC3* has a higher propensity for founder mutations (due to particular geographical and cultural isolation)

Interestingly, by comparing the distribution of variants in our region of Central-Europe, we found very few overlaps (with regard P/LP variants). Only two variants, reported in the Polish cohort [14], were identified in our cohort (*MYH7* p. Glu1039Gly and *MYBPC3* p.Arg495Trp). Most notably, the variant *MYBPC3* p.Tyr847Ter, reported to be a likely founder mutation in the Polish population, was not detected in our patient cohort. In the Slovak cohort [12], only two identical variants were identified (*MYH7* p.Arg663His and *MYBPC3* p.Tyr1136del). With regard to the Romanian cohort [13], only the *TNNI3* p.Arg186Gln was present in our patient group. With the Czech HCM [15] cohort, there were eight overlapping variants (*MYBPC3* p.Arg495Gln, p.Arg810His, p.Ala1056GlyfsTer9, and p.Gln1233Ter; *MYH7* p.Ile736Thr and p.Glu924Lys; *MYL3* p.Ala57Gly; and *TNNI3* p.Ser166Phe). Out of the three Hungarian putative founder variants, only the *MYBPC3* p.Gln1233Ter mutation was identified in one patient in the Czech cohort.

A total of 63% of our HCM cohort proved to be likely genotype-negative cases. The etiology of genotype-negative HCM may include rare variants in yet unknown genes or in regulatory non-coding regions of known causative genes [33]. Such genes, like *TRIM63* [34], *ALPK3* [35], or *FHOD3* [36], were indeed reported but as being responsible only for 1–2% of genotype-negative cases. Another possible explanation may also be the presence of variants affecting cryptic splice-altering sites, which have also been reported in *MYBPC3* in 2.2% of gene-negative patients [37]. Recently, data on the polygenic nature of HCM has emerged with a surprisingly high degree of possible polygenic contribution of the disease [38,39].

In conclusion, similar to other HCM populations, the *MYBPC3* and *MYH7* genes seem to be the most commonly affected genes in Hungarian HCM patients. The high prevalence of three *MYBPC3* mutations raises the possibility of a founder effect in our HCM cohort.

## 5. Study Limitations

Although this was a nationwide study, the participating centers are all third-level referral cardiology institutions with a possible bias towards accumulating more severe or problematic cases. Furthermore, even with considerable development regarding the evaluation of different gene variants, the interpretation of gene variants still remained somehow individual. Although we applied a very strict interpretation process, based on the user-independent CardioClassifier program and the ClinVar database, both of these partly depend on the number of already-reported clinical cases associated with the given genetic variant. The latter might have influenced the variant interpretation, especially that of the ‘variant of unknown significance’ category. Comparisons of our results with those obtained in other Central-European populations may be influenced also by the relatively small number of patients assessed in the respective populations.

## Figures and Tables

**Table 1:** Main demographic, clinical, and echocardiographic characteristics of the studied Hungarian index HCM patients (n = 242). Data are expressed as mean value ± SD or number (%) of patients. * In patients with a resting gradient >50 mmHg. Abbreviations: HCM: hypertrophic cardiomyopathy; ICD: implantable cardioverter defibrillator; LV: left ventricular; LVOTG: left ventricular outflow tract gradient; PTSMA: percutaneous transluminal septal myocardial ablation.

**Demographics**	
Males:	127 (52%)
Age at diagnosis (years):	44 ± 11
Follow up (years):	12 ± 9
Familial disease (proven):	62 (25.5%)
**Echocardiography data**	
Left atrial diameter (mm):	46 ± 10
Maximal LV wall thickness (mm):	23 ± 7
LV end-diastolic diameter (mm):	44 ± 13
LV end-systolic diameter (mm):	26 ± 11
LV ejection fraction (%):	68 ± 13
LVOTG rest ≥ 50 mm Hg:	49 (20%)
LVOTG rest (mmHg) *:	74 ± 36 Hgmm
LVOTG Valsalva (mmHg) *:	93 ± 48 Hgmm
**Treatment, device, and intervention**	
Medical treatment:	
Beta blocker:	230 (95%)
Verapamil:	17 (7%)
Disopyramide:	50 (20%)
Amiodarone:	41 (17%)
ICD implantation:	46 (19%)
Septal reduction therapy (PTSMA or myectomy):	32 (13%)
**Follow up:**	
Death during follow up:	43 (18%)
Age at death (years):	52 ± 12
Death after diagnosis (years):	10 ± 7
HCM-related death:	32 (75%)
**Cause of death:**	
Heart failure:	10 (25%)
Sudden cardiac death:	12 (28%)
Thromboembolism:	6 (13%)
Other:	4 (9%)

**Table 2:** Patient-level variant analysis, with the number (n) and percentage (%) of the identified variants in the different genes. Definitive: genes with definitive evidence for HCM association; moderate: genes with moderate evidence for HCM association; P/LP: pathogenic/likely pathogenic variant; VUS: variant of unknown significance; LVH: left ventricular hypertrophy. * in patients without P/LP variants.

		P/LP, n (%)	VUS * n (%)
Definitive	*MYBPC3*	55 (61)	9 (20)
	*MYH7*	21 (23)	11 (24)
	*TNNT2*	3 (3)	2 (4)
	*TNNI3*	3 (3)	1 (2)
	*TPM1*	1 (1)	4 (9)
	*ACTC1*	1 (1)	2 (4)
	*MYL2*	0 (0)	3 (7)
	*MYL3*	0 (0)	3 (7)
Moderate	*CSRP3*	0 (0)	3 (7)
	*TNNC1*	0 (0)	1 (2)
	*JPH2*	0 (0)	0 (0)
Syndromic genes with isolated LVH	*DES*	1 (1)	1 (2)
	*FHL1*	1 (1)	1 (2)
	*RAF1*	2 (2)	0 (0)
	*PRKAG2*	0 (0)	4 (9)
	*PTPN11*	0 (0)	1 (2)
Other	mitochondrial	1 (1)	NA
	*TTN*	1 (1)	NA
	**Total**	**90 (100)**	**46 (100)**

**Table 3:** Gene-level variant analysis, with the number (n) and the percentage (%) of variants identified in different genes. Definitive: genes with definitive evidence for HCM association; moderate: genes with moderate evidence for HCM association; P/LP: pathogenic/likely pathogenic variant; VUS: variant of unknown significance; B/LB: benign/likely benign variant; LVH: left ventricular hypertrophy.

		P/LP, n (%)	VUS, n (%)	B/LB, n (%)	Total, n (%)
Definitive	*MYBPC3*	22 (59)	9 (24)	6 (16)	37 (100)
	*MYH7*	17 (57)	12 (40)	1 (3)	30 (100)
	*TNNT2*	2 (33)	4 (67)	0 (0)	6 (100)
	*TNNI3*	3 (60)	2 (40)	0 (0)	5 (100)
	*TPM1*	1 (20)	4 (80)	0 (0)	5 (100)
	*ACTC1*	1 (33)	2 (67)	0 (0)	3 (100)
	*MYL2*	0 (0)	4 (100)	0 (0)	4 (100)
	*MYL3*	0 (0)	4 (100)	0 (0)	4 (100)
Moderate	*CSRP3*	0 (0)	3 (75)	1 (25)	4 (100)
	*TNNC1*	0 (0)	1 (100)	0 (0)	1 (100)
	*JPH2*	0 (0)	4 (67)	2 (33)	6 (100)
Syndromic genes with isolated LVH	*ACTN2*	0 (0)	2 (50)	2 (50)	4 (100)
	*DES*	1 (14)	5 (71)	1 (14)	7 (100)
	*FHL1*	1 (50)	1 (50)	0 (0)	2 (100)
	*RAF1*	2 (100)	0 (0)	0 (0)	2 (100)
	*PRKAG2*	0 (0)	3 (75)	1 (25)	4 (100)
	*LAMP2*	0 (0)	0 (0)	3 (100)	3 (100)
	*PTPN11*	0 (0)	1 (100)	0 (0)	1 (100)
Other	mitochondrial	1 (100)	NA	NA	1 (100)
	*TTN*	1 (100)	NA	NA	1 (100)

## Data Availability

To ensure independent interpretation of the study results, the authors grant all external authors access to relevant material, including participant-level clinical study data. Study documents and participant clinical study data are available to be shared on request after publication of the primary manuscript. Bona fide, qualified scientific and medical researchers are eligible to request access to the clinical study data with corresponding documentation describing the structure and content of the datasets. Data are shared in a secure data-access system. Prior to providing access, clinical study documents and data will be examined, and, if necessary, redacted and de-identified to protect the personal data of the study participants and personnel, and to respect the boundaries of the informed consent of the study participants.

## Supplementary Materials

The following supporting information can be downloaded at: https://www.mdpi.com/article/10.3390/diagnostics12051132/s1. Table S1. Genes included in the panel for targeted resequencing. Table S2. Complete list and attributes of the variants identified in the study. The mitochondrial mutation is not included. Table S3. Gene-level variant analysis, with the number (n) and the percentage (%) of variants identified in different genes [percentage is given as per class of variants (i.e. per P/LP, per VUS, per B/LB variants)].

## References

[1] Elliott P.M., Anastasakis A., Borger M.A., Borggrefe M., Cecchi F., Charron P., Hagege A.A., Lafont A., Limongelli G., Mahrholdt H. (2014). 2014 ESC Guidelines on diagnosis and management of hypertrophic cardiomyopathy: The Task Force for the Diagnosis and Management of Hypertrophic Cardiomyopathy of the European Society of Cardiology (ESC). Eur. Heart J..

[2] Semsarian C., Ingles J., Maron M.S., Maron B.J. (2015). New perspectives on the prevalence of hypertrophic cardiomyopathy. J. Am. Coll. Cardiol..

[3] Maron B.J., Gardin J.M., Flack J.M., Gidding S.S., Kurosaki T.T., Bild D.E. (1995). Prevalence of hypertrophic cardiomyopathy in a general population of young adults. Echocardiographic analysis of 4111 subjects in the CARDIA Study. Coronary Artery Risk Development in (Young) Adults. Circulation.

[4] Geisterfer-Lowrance A.A.T., Kass S., Tanigawa G., Vosberg H.-P., McKenna W.J., Seidman C.E., Seidman J.G. (1990). A molecular basis for familial hypertrophic cardiomyopathy: A beta cardiac myosin heavy chain gene missense mutation. Cell.

[5] Bonne G., Carrier L., Bercovici J., Cruaud C., Richard P., Hainque B., Gautel M., Labeit S., James M., Beckmann J. (1995). Cardiac myosin binding protein-C gene splice acceptor site mutation is associated with familial hypertrophic cardiomyopathy. Nat. Genet..

[6] Kimura A., Harada H., Park J.-E., Nishi H., Satoh M., Takahashi M., Hiroi S., Sasaoka T., Ohbuchi N., Nakamura T. (1997). Mutations in the troponin I gene associated with hypertrophic cardiomyopathy. Nat. Genet..

[7] Mogensen J., Klausen I., Pedersen A., Egeblad H., Bross P., Kruse T., Gregersen N., Hansen P., Baandrup U., Borglum A. (1999). Alpha-cadiac actin is a novel disease gene in familial hypertrophic cardiomyopathy. J. Clin. Investig..

[8] Poetter K., Jiang H., Hassanzadeh S., Master S.R., Chang A., Dalakas M.C., Rayment I., Sellers J.R., Fananapazir L., Epstein N.D. (1996). Mutations in either the essential or regulatory light chains of myosin are associated with a rare myopathy in human heart and skeletal muscle. Nat. Genet..

[9] Thierfelder L., Watkins H., MacRae C., Lamas R., McKenna W., Vosberg H.P., Seidman J.G., Seidman C.E. (1994). Alpha-tropomyosin and cardiac troponin T mutations cause familial hypertrophic cardiomyopathy: A disease of the sarcomere. Cell.

[10] Watkins H., Conner D., Thierfelder L., Jarcho J.A., MacRae C., McKenna W.J., Maron B.J., Seidman J.G., Seidman C.E. (1995). Mutations in the cardiac myosin binding protein-C gene on chromosome 11 cause familial hypertrophic cardiomyopathy. Nat. Genet..

[11] Ommen S.R., Mital S., Burke M.A., Day S.M., Deswal A., Elliott P., Evanovich L.L., Hung J., Joglar J.A., Kantor P. (2020). 2020 AHA/ACC Guideline for the Diagnosis and Treatment of Patients With Hypertrophic Cardiomyopathy: Executive Summary: A Report of the American College of Cardiology/American Heart Association Joint Committee on Clinical Practice Guidelines. Circulation.

[12] Nagyova E., Radvanszky J., Hyblova M., Simovicova V., Goncalvesova E., Asselbergs F.W., Kadasi L., Szemes T., Minarik G. (2019). Targeted next-generation sequencing in Slovak cardiomyopathy patients. Bratisl. Med. J..

[13] Micheu M.M., Popa-Fotea N.M., Oprescu N., Bogdan S., Dan M., Deaconu A., Dorobantu L., Gheorghe-Fronea O., Greavu M., Iorgulescu C. (2020). Yield of Rare Variants Detected by Targeted Next-Generation Sequencing in a Cohort of Romanian Index Patients with Hypertrophic Cardiomyopathy. Diagnostics.

[14] Lipari M., Wypasek E., Karpinski M., Tomkiewicz-Pajak L., Laino L., Binni F., Giannarelli D., Rubis P., Petkow-Dimitrow P., Undas A. (2020). Identification of a variant hotspot in MYBPC3 and of a novel CSRP3 autosomal recessive alteration in a cohort of Polish patients with hypertrophic cardiomyopathy. Pol. Arch. Intern. Med..

[15] Bonaventura J., Norambuena P., Tomasov P., Jindrova D., Sediva H., Macek M., Veselka J. (2019). The utility of the Mayo Score for predicting the yield of genetic testing in patients with hypertrophic cardiomyopathy. Arch. Med. Sci..

[16] Post H., Nemeth E., Klima L., Flores R., Feher T., Turk A., Szekely G., Sahakyan H., Mondal M., Montinaro F. (2019). Y-chromosomal connection between Hungarians and geographically distant populations of the Ural Mountain region and West Siberia. Sci. Rep..

[17] Ingles J., Goldstein J., Thaxton C., Caleshu C., Corty E.W., Crowley S.B., Dougherty K., Harrison S.M., McGlaughon J., Milko L.V. (2019). Evaluating the Clinical Validity of Hypertrophic Cardiomyopathy Genes. Circ. Genom. Precis. Med..

[18] Richards S., Aziz N., Bale S., Bick D., Das S., Gastier-Foster J., Grody W.W., Hegde M., Lyon E., Spector E. (2015). Standards and guidelines for the interpretation of sequence variants: A joint consensus recommendation of the American College of Medical Genetics and Genomics and the Association for Molecular Pathology. Genet. Med..

[19] Kelly M.A., Caleshu C., Morales A., Buchan J., Wolf Z., Harrison S.M., Cook S., Dillon M.W., Garcia J., Haverfield E. (2018). Adaptation and validation of the ACMG/AMP variant classification framework for MYH7-associated inherited cardiomyopathies: Recommendations by ClinGen’s Inherited Cardiomyopathy Expert Panel. Genet. Med..

[20] Whiffin N., Walsh R., Govind R., Edwards M., Ahmad M., Zhang X., Tayal U., Buchan R., Midwinter W., Wilk A.E. (2018). CardioClassifier: Disease- and gene-specific computational decision support for clinical genome interpretation. Genet. Med..

[21] Parfait B., Chretien D., Rotig A., Marsac C., Munnich A., Rustin P. (2000). Compound heterozygous mutations in the flavoprotein gene of the respiratory chain complex II in a patient with Leigh syndrome. Hum. Genet..

[22] Alfares A.A., Kelly M.A., McDermott G., Funke B.H., Lebo M.S., Baxter S.B., Shen J., McLaughlin H.M., Clark E.H., Babb L.J. (2015). Results of clinical genetic testing of 2912 probands with hypertrophic cardiomyopathy: Expanded panels offer limited additional sensitivity. Genet. Med..

[23] Bos J.M., Will M.L., Gersh B.J., Kruisselbrink T.M., Ommen S.R., Ackerman M.J. (2014). Characterization of a phenotype-based genetic test prediction score for unrelated patients with hypertrophic cardiomyopathy. Mayo Clin. Proc..

[24] Gomez J., Reguero J.R., Moris C., Martin M., Alvarez V., Alonso B., Iglesias S., Coto E. (2014). Mutation analysis of the main hypertrophic cardiomyopathy genes using multiplex amplification and semiconductor next-generation sequencing. Circ. J..

[25] Hathaway J., Helio K., Saarinen I., Tallila J., Seppala E.H., Tuupanen S., Turpeinen H., Kangas-Kontio T., Schleit J., Tommiska J. (2021). Diagnostic yield of genetic testing in a heterogeneous cohort of 1376 HCM patients. BMC Cardiovasc. Disord..

[26] Jaaskelainen P., Vangipurapu J., Raivo J., Kuulasmaa T., Helio T., Aalto-Setala K., Kaartinen M., Ilveskoski E., Vanninen S., Hamalainen L. (2019). Genetic basis and outcome in a nationwide study of Finnish patients with hypertrophic cardiomyopathy. ESC Heart Fail..

[27] Lopes L.R., Zekavati A., Syrris P., Hubank M., Giambartolomei C., Dalageorgou C., Jenkins S., McKenna W., Plagnol V., Uk10k Consortium (2013). Genetic complexity in hypertrophic cardiomyopathy revealed by high-throughput sequencing. J. Med. Genet..

[28] Walsh R., Buchan R., Wilk A., John S., Felkin L.E., Thomson K.L., Chiaw T.H., Loong C.C.W., Pua C.J., Raphael C. (2017). Defining the genetic architecture of hypertrophic cardiomyopathy: Re-evaluating the role of non-sarcomeric genes. Eur. Heart J..

[29] Erdmann J., Daehmlow S., Wischke S., Senyuva M., Werner U., Raible J., Tanis N., Dyachenko S., Hummel M., Hetzer R. (2003). Mutation spectrum in a large cohort of unrelated consecutive patients with hypertrophic cardiomyopathy. Clin. Genet..

[30] Ingles J., Doolan A., Chiu C., Seidman J., Seidman C., Semsarian C. (2005). Compound and double mutations in patients with hypertrophic cardiomyopathy: Implications for genetic testing and counselling. J. Med. Genet..

[31] Christiaans I., Nannenberg E.A., Dooijes D., Jongbloed R.J., Michels M., Postema P.G., Majoor-Krakauer D., van den Wijngaard A., Mannens M.M., van Tintelen J.P. (2010). Founder mutations in hypertrophic cardiomyopathy patients in the Netherlands. Neth. Heart J..

[32] Jaaskelainen P., Helio T., Aalto-Setala K., Kaartinen M., Ilveskoski E., Hamalainen L., Melin J., Nieminen M.S., Laakso M., Kuusisto J. (2013). Two founder mutations in the alpha-tropomyosin and the cardiac myosin-binding protein C genes are common causes of hypertrophic cardiomyopathy in the Finnish population. Ann. Med..

[33] Bagnall R.D., Ingles J., Dinger M.E., Cowley M.J., Ross S.B., Minoche A.E., Lal S., Turner C., Colley A., Rajagopalan S. (2018). Whole Genome Sequencing Improves Outcomes of Genetic Testing in Patients With Hypertrophic Cardiomyopathy. J. Am. Coll. Cardiol..

[34] Salazar-Mendiguchia J., Ochoa J.P., Palomino-Doza J., Dominguez F., Diez-Lopez C., Akhtar M., Ramiro-Leon S., Clemente M.M., Perez-Cejas A., Robledo M. (2020). Mutations in TRIM63 cause an autosomal-recessive form of hypertrophic cardiomyopathy. Heart.

[35] Almomani R., Verhagen J.M., Herkert J.C., Brosens E., van Spaendonck-Zwarts K.Y., Asimaki A., van der Zwaag P.A., Frohn-Mulder I.M., Bertoli-Avella A.M., Boven L.G. (2016). Biallelic Truncating Mutations in ALPK3 Cause Severe Pediatric Cardiomyopathy. J. Am. Coll. Cardiol..

[36] Ochoa J.P., Sabater-Molina M., Garcia-Pinilla J.M., Mogensen J., Restrepo-Cordoba A., Palomino-Doza J., Villacorta E., Martinez-Moreno M., Ramos-Maqueda J., Zorio E. (2018). Formin Homology 2 Domain Containing 3 (FHOD3) Is a Genetic Basis for Hypertrophic Cardiomyopathy. J. Am. Coll. Cardiol..

[37] Lopes L.R., Barbosa P., Torrado M., Quinn E., Merino A., Ochoa J.P., Jager J., Futema M., Carmo-Fonseca M., Monserrat L. (2020). Cryptic Splice-Altering Variants in MYBPC3 Are a Prevalent Cause of Hypertrophic Cardiomyopathy. Circ. Genom. Precis. Med..

[38] Harper A.R., Goel A., Grace C., Thomson K.L., Petersen S.E., Xu X., Waring A., Ormondroyd E., Kramer C.M., Ho C.Y. (2021). Common genetic variants and modifiable risk factors underpin hypertrophic cardiomyopathy susceptibility and expressivity. Nat. Genet..

[39] Tadros R., Francis C., Xu X., Vermeer A.M.C., Harper A.R., Huurman R., Kelu Bisabu K., Walsh R., Hoorntje E.T., Te Rijdt W.P. (2021). Shared genetic pathways contribute to risk of hypertrophic and dilated cardiomyopathies with opposite directions of effect. Nat. Genet..

