# Supplementary material for: The Genetic Architecture of Hypertrophic Cardiomyopathy in Hungary: Analysis of 242 Patients with a Panel of 98 Genes
Source: Diagnostics (Basel). 2022 May 3;12(5):1132. doi: 10.3390/diagnostics12051132 (PMC9139509; doi:10.3390/diagnostics12051132)
Supplement: Supplementary file 1 [file diagnostics-12-01132-s001.zip › diagnostics-1699952-supplementary.pdf]

Supplementary Tables for the article:

Róbert Sepp, Lidia Hategan, Beáta Csányi, János Borbás, Annamária Tringer, Eszter Dalma Pálinkás, Viktória Nagy, Hedvig Takács, Dóra Latinovics, Noémi Nyolczas, Attila Pálinkás, Réka Faludi, Miklós Rábai, Tamás Gábor Szabó, Dániel Czuriga, László Balogh, Róbert Halmosi, Attila Borbély, Tamás Habon, Zoltán Hegedűs, István Nagy. The genetic architecture of hypertrophic cardiomyopathy in Hungary: analysis of 242 patients with a panel of 98 genes

Supplementary Table S1. **Genes included in the panel for targeted resequencing.**

Supplementary Table S2. **Complete list and attributes of the variants identified in the study. The mitochondrial mutation is not included.**

Supplementary Table S3. Gene-level variant analysis, with the number (n) and the percentage (%) of variants identified in different genes [percentage is given as per class of variants (i.e. per P/LP, per VUS, per B/LB variants)]. Definitive: genes with definitive evidence for HCM association; moderate: genes with moderate evidence for HCM association; P/LP: pathogenic/likely pathogenic variant; VUS: variant of unknown significance; B/LB: benign/likely benign variant; LVH: left ventricular hypertrophy.

Supplementary Table S1. **Genes included in the panel for targeted resequencing.**

GENE SYMBOL	GENE NAME	ENSG NUMBER
**Genes with definitive evidence for HCM association**		
MYBPC3	myosin binding protein C3	ENSG00000134571
MYH7	myosin heavy chain 7	ENSG0000009205
TNNT2	troponin T2, cardiac type	ENSG00000118194
TNNI3	troponin I3, cardiac type	ENSG00000129991
TPM1	tropomyosin 1	ENSG00000140416
ACTC1	actin alpha cardiac muscle 1	ENSG00000159251
MYL2	myosin light chain 2	ENSG00000111245
MYL3	myosin light chain 3	ENSG00000160808
**Genes with moderate evidence for HCM association**		
CSRP3	cysteine and glycine rich protein 3	ENSG00000129170
JPH2	junctophilin 2	ENSG00000149596
TNNC1	troponin C1, slow skeletal and cardiac type	ENSG00000114854

**Intrinsic cardiomyopathy genes**		
ACTN2	actinin alpha 2	ENSG00000077522
PLN	phospholamban	ENSG00000198523
**Syndromic genes, where isolated left ventricular hypertrophy (LVH) may be seen**		
DES	desmin	ENSG00000175084
FHL1	four and a half LIM domains 1	ENSG00000022267
LAMP2	lysosomal associated membrane protein 2	ENSG00000005893
PRKAG2	protein kinase AMP-activated non-catalytic subunit gamma 2	ENSG00000106617
PTPN11	protein tyrosine phosphatase non-receptor type 11	ENSG00000179295
RAF1	Raf-1 proto-oncogene, serine/threonine kinase	ENSG00000132155
TTR	transthyretin	ENSG00000118271
**Genes with definitive or moderate evidence for DCM or ARVC association**		
BAG3	BAG cochaperone 3	ENSG00000151929
LMNA	lamin A/C	ENSG00000160789
RBM20	RNA binding motif protein 20	ENSG00000203867

TTN	titin	ENSG00000155657
DSP	desmoplakin	ENSG00000096696
PKP2	plakophilin 2	ENSG00000057294
DSG2	desmoglein 2	ENSG00000046604
DSC2	desmocollin 2	ENSG00000134755
JUP	junction plakoglobin	ENSG00000173801
TMEM43	transmembrane protein 43	ENSG00000170876
**Genes with limited evidence for HCM association**		
MYPN	myopalladin	ENSG00000138347
ANKRD1	ankyrin repeat domain 1	ENSG00000148677
MYLK2	myosin light chain kinase 2	ENSG00000101306
MYOZ2	myozenin 2	ENSG00000172399
NEXN	nexilin F-actin binding protein	ENSG00000162614
VCL	vinculin	ENSG00000035403
TRIM63	tripartite motif containing 63	ENSG00000158022
MYH6	myosin heavy chain 6	ENSG00000197616

PDLIM3	PDZ and LIM domain 3	ENSG00000154553
TCAP	titin-cap	ENSG00000173991
MYOM1	myomesin 1	ENSG00000101605
CALR3	calreticulin 3	ENSG00000269058
**Genes with limited evidence for DCM or ARVC association**		
ABCC9	ATP binding cassette subfamily C member 9	ENSG00000069431
CTF1	cardiotrophin 1	ENSG00000150281
DTNA	dystrobrevin alpha	ENSG00000134769
EYA4	EYA transcriptional coactivator and phosphatase 4	ENSG00000112319
GATAD1	GATA zinc finger domain containing 1	ENSG00000157259
ILK	integrin linked kinase	ENSG00000166333
LAMA4	laminin subunit alpha 4	ENSG00000112769
LDB3	LIM domain binding 3	ENSG00000122367
NEBL	nebulette	ENSG00000078114
PSEN2	presenilin 2	ENSG00000143801
SGCD	sarcoglycan delta	ENSG00000170624

**Syndromic genes, where LVH is seen with overt syndromic features**		
CAV3	caveolin 3	ENSG00000182533
CRYAB	crystallin alpha B	ENSG00000109846
FXN	frataxin	ENSG00000165060
GAA	alpha glucosidase	ENSG00000171298
**Other genes**		
ACADVL	acyl-CoA dehydrogenase very long chain	ENSG00000072778
AGL	amylo-alpha-1, 6-glucosidase, 4-alpha-glucanotransferase	ENSG00000162688
ATP5E	ATP synthase F1 subunit epsilon	ENSG00000124172
BRAF	B-Raf proto-oncogene, serine/threonine kinase	ENSG00000157764
CASQ2	calsequestrin 2	ENSG00000118729
CBL	Cbl proto-oncogene	ENSG00000110395
COA5	cytochrome c oxidase assembly factor 5	ENSG00000183513
CTNNA3	catenin alpha 3	ENSG00000183230
DMD	dystrophin	ENSG00000198947
DMPK	DM1 protein kinase	ENSG00000104936

DNAJC19	DnaJ heat shock protein family (Hsp40) member C19	ENSG00000205981
DNM1L	dynamin 1 like	ENSG00000087470
DOLK	dolichol kinase	ENSG00000175283
EMD	emerin	ENSG00000102119
FHL2	four and a half LIM domains 2	ENSG00000115641
FKTN	fukutin	ENSG00000106692
FOXRED1	FAD dependent oxidoreductase domain containing 1	ENSG00000110074
GUSB	glucuronidase beta	ENSG00000169919
HFE	homeostatic iron regulator	ENSG00000010704
HRAS	HRas proto-oncogene, GTPase	ENSG00000174775
KRAS	KRAS proto-oncogene, GTPase	ENSG00000133703
MAP2K1	mitogen-activated protein kinase kinase 1	ENSG00000169032
MAP2K2	mitogen-activated protein kinase kinase 2	ENSG00000126934
MIB1	MIB E3 ubiquitin protein ligase 1	ENSG00000101752
MRPL3	mitochondrial ribosomal protein L3	ENSG00000114686
NRAS	NRAS proto-oncogene, GTPase	ENSG00000213281

PSEN1	presenilin 1	ENSG00000080815
SCO2	synthesis of cytochrome C oxidase 2	ENSG00000284194
SDHA	succinate dehydrogenase complex flavoprotein subunit A	ENSG00000073578
SHOC2	SHOC2 leucine rich repeat scaffold protein	ENSG00000108061
SLC25A3	solute carrier family 25 member 3	ENSG00000075415
SOS1	SOS Ras/Rac guanine nucleotide exchange factor 1	ENSG00000115904
SPRED1	sprouty related EVH1 domain containing 1	ENSG00000166068
SYNE1	spectrin repeat containing nuclear envelope protein 1	ENSG00000131018
SYNE2	spectrin repeat containing nuclear envelope protein 2	ENSG00000054654
TAZ	ZNF561 antisense RNA 1 (head to head)	ENSG00000267106
TGFB3	transforming growth factor beta 3	ENSG00000119699
TMEM70	transmembrane protein 70	ENSG00000175606
TMPO	thymopoietin	ENSG00000120802
TSFM	Ts translation elongation factor, mitochondrial	ENSG00000123297
TXNRD2	thioredoxin reductase 2	ENSG00000184470

Supplementary Table S2. **Complete list and attributes of the variants identified in the study. The mitochondrial mutation is not included.**

Gene	Variant type	coding HGVS	protein HGVS	ENST	ENSP	Final verdict	Plus items for final verdict	Cardio Classifier	Cardio Classifier items	ClinVar max	ClinVar score	ClinVar entry, n	Novel
ACTC1													
ACTC1	missense	c.281A>T	p.Asn94Ile	ENST00000290378:c.281A>T	ENSP00000290378:p.Asn94Ile	pathogenic	PS4, PP1	VUS	PM2, PP2, PP3	4	4	1	no
ACTC1	missense	c.67T>C	p.Phe23Leu	ENST00000290378:c.67T>C	ENSP00000290378:p.Phe23Leu	VUS	-	VUS	PM2, PP2, PP3	4	3,3	3	no
ACTC1	missense	c.698C>T	p.Ala233Val	ENST00000290378:c.698C>T	ENSP00000290378:p.Ala233Val	VUS	-	VUS	PM2, PP2, PP3	-	-	-	yes
ACTN2													
ACTN2	missense	c.2677G>A	p.Asp893Asn	ENST00000366578.6:c.2677G>A	ENSP00000355537.4:p.Asp893Asn	VUS	-	-	-	3	2,67	3	no
ACTN2	missense	c.893G>A	p.Arg298His	ENST00000366578.6:c.893G>A	ENSP00000355537.4:p.Arg298His	likely benign	-	-	-	3	2,36	11	no
ACTN2	missense	c.1930G>A	p.Ala644Thr	ENST00000366578:c.1930G>A	ENSP00000355537:p.Ala644Thr	VUS	-	-	-	3	3	8	no
ACTN2	missense	c.2147C>T	p.Thr716Met	ENST00000366578:c.2147C>T	ENSP00000355537:p.Thr716Met	likely benign	-	-	-	3	2,28	14	no
CSRP3													
CSRP3	missense	c.10T>C	p.Trp4Arg	ENST00000533783:c.10T>C	ENSP00000431813:p.Trp4Arg	benign	-	benign	-	3	1,91	12	no
CSRP3	missense	c.206A>G	p.Lys69Arg	ENST00000533783:c.206A>G	ENSP00000431813:p.Lys69Arg	VUS	-	VUS	PM2, PP3	3	3	5	no
CSRP3	missense	c.136A>C	p.Ser46Arg	ENST00000533783:c.136A>C	ENSP00000431813:p.Ser46Arg	VUS	-	VUS	PM2, PP3	5	3,4	5	no
CSRP3	missense	c.208G>T	p.Gly70Trp	ENST00000533783:c.208G>T	ENSP00000431813:p.Gly70Trp	VUS	-	VUS	PP3	3	3	3	no
DES													
DES	missense	c.1216C>T	p.Arg406Trp	ENST00000373960:c.1216C>T	ENSP00000363071:p.Arg406Trp	pathogenic	-	-	-	5	4,9	10	no
DES	missense	c.1286G>A	p.Arg429Gln	ENST00000373960:c.1286G>A	ENSP00000363071:p.Arg429Gln	VUS	-	-	-	3	3	4	no
DES	missense	c.206T>C	p.Leu69Pro	ENST00000373960:c.206T>C	ENSP00000363071:p.Leu69Pro	VUS	-	-	-	-	-	-	yes
DES	missense	c.250G>A	p.Gly84Ser	ENST00000373960:c.250G>A	ENSP00000363071:p.Gly84Ser	VUS	-	-	-	3	2,89	9	no
DES	missense	c.569T>G	p.Leu190Arg	ENST00000373960:c.569T>G	ENSP00000363071:p.Leu190Arg	VUS	-	-	-	-	-	-	yes
DES	missense	c.638C>T	p.Ala213Val	ENST00000373960:c.638C>T	ENSP00000363071:p.Ala213Val	benign	-	-	-	3	1,44	16	no
DES	missense	c.728A>G	p.His243Arg	ENST00000373960:c.728A>G	ENSP00000363071:p.His243Arg	VUS	-	-	-	3	3	4	no

FHL1													
FHL1	missense	c.283C>T	p.Arg95Trp	ENST00000370674.3:c.283C>T	ENSP00000359708.1:p.Arg95Trp	VUS	-	VUS	BS1, PP3, PP2	2	1,67	3	no
FHL1	nonsense	c.286G>T	p.Glu96*	ENST00000370690:c.286G>T	ENSP00000359724:p.Glu96*	likely pathogenic	-	likely pathogenic	PVS1, PM2	-	-	-	yes
JPH2													
JPH2	missense	c.1522A>C	p.Ser508Arg	ENST00000372980:c.1522A>C	ENSP00000362071:p.Ser508Arg	VUS	-	-	-	-	-	-	yes
JPH2	missense	c.1801A>C	p.Thr601Pro	ENST00000372980:c.1801A>C	ENSP00000362071:p.Thr601Pro	VUS	-	-	-	-	-	-	yes
JPH2	missense	c.281A>G	p.Tyr94Cys	ENST00000372980:c.281A>G	ENSP00000362071:p.Tyr94Cys	VUS	-	-	-	-	-	-	yes
JPH2	missense	c.572C>G	p.Pro191Arg	ENST00000372980:c.572C>G	ENSP00000362071:p.Pro191Arg	likely benign	-	-	-	3	1,8	10	no
JPH2	missense	c.605T>G	p.Leu202Arg	ENST00000372980:c.605T>G	ENSP00000362071:p.Leu202Arg	VUS	-	-	-	-	-	-	yes
JPH2	missense	c.856A>G	p.Thr286Ala	ENST00000372980:c.856A>G	ENSP00000362071:p.Thr286Ala	likely benign	-	-	-	2	2	5	no
LAMP2													
LAMP2	intronic variant	c.1093+2514G>A	-	ENST00000200639:c.1093+2514G>A	-	benign	-	-	-	3	1,46	13	no
LAMP2	missense	c.661G>A	p.Gly221Arg	ENST00000200639:c.661G>A	ENSP00000200639:p.Gly221Arg	benign	-	benign	ExAC frequency	3	1,58	11	no
LAMP2	missense	c.755T>G	p.Ile252Ser	ENST00000200639:c.755T>G	ENSP00000200639:p.Ile252Ser	benign	-	benign	ExAC frequency	2	1,54	13	no
MYBPC3													
MYBPC3	missense	c.1483C>T	p.Arg495Trp	ENST00000545968.6:c.1483C>T	ENSP00000442795.1:p.Arg495Trp	likely pathogenic	-	likely pathogenic	PM2, PM5, PP2, PP3	5	4,5	8	no
MYBPC3	missense	c.1484G>A	p.Arg495Gln	ENST00000545968.6:c.1484G>A	ENSP00000442795:p.Arg495Gln	pathogenic	PM5	likely pathogenic	PS4, PM2, PP1, PP2	5	4,8	22	no
MYBPC3	frameshift	c.1776_1777delGT	p.Ser593ProfsTer11	ENST00000545968.6:c.1776_1777del	ENSP00000442795.1:p.Ser593ProfsTer11	pathogenic	PS4, PP1	likely pathogenic	PVS1, PM2	5	5	4	no
MYBPC3	missense	c.1855G>A	p.Glu619Lys	ENST00000545968.6:c.1855G>A	ENSP00000442795:p.Glu619Lys	benign	-	benign	ExAC frequency	3	2	16	no
MYBPC3	nonsense	c.2048G>A	p.Trp683X	ENST00000545968.6:c.2048G>A	ENSP00000442795:p.Trp683*	likely pathogenic	-	likely pathogenic	PVS1, PM2	5	4,6	3	no
MYBPC3	frameshift	c.2373dupG	p.Trp792ValfsTer41	ENST00000545968.6:c.2373dup	ENSP00000442795:p.Trp792fs	pathogenic	-	pathogenic	PVS1, PS4, PM2	5	5	24	no
MYBPC3	missense	c.2429G>A	p.Arg810His	ENST00000545968.6:c.2429G>A	ENSP00000442795:p.Arg810His	likely pathogenic	-	likely pathogenic	PS4, PM2, PP2, PP1	4	3,4	9	no
MYBPC3	missense	c.2459G>C	p.Arg820Pro	ENST00000545968.6:c.2459G>C	ENSP00000442795.1:p.Arg820Pro	VUS	-	VUS	PM2, PP2	3	3	1	no
MYBPC3	missense	c.2543C>A	p.Ala848Glu	ENST00000545968.6:c.2543C>A	ENSP00000442795.1:p.Ala848Glu	VUS	-	VUS	PM2, PP2, PP3	3	3	3	no

MYBPC3	nonsense	c.2827C>T	p.Arg943X	ENST00000545968.6:c.2827C>T	ENSP00000442795:p.Arg943*	pathogenic	-	pathogenic	PVS1, PS4, PP1, PM2	5	5	17	no
MYBPC3	frameshift	c.2864_2865delCT	p.Pro955ArgfsTer95	ENST00000545968.6:c.2864_2865del	ENSP00000442795.1:p.Pro955ArgfsTer	pathogenic	-	pathogenic	PVS1, PS3, PS4, PP1, PM2	5	4,7	18	no
MYBPC3	missense	c.2870C>G	p.Thr957Ser	ENST00000545968.6:c.2870C>G	ENSP00000442795.1:p.Thr957Ser	benign	-	benign	ExAC frequency	3	2,3	18	no
MYBPC3	missense	c.2873C>T	p.Thr958Ile	ENST00000545968.6:c.2873C>T	ENSP00000442795.1:p.Thr958Ile	VUS	-	VUS	PP2, BP4	3	2,8	13	no
MYBPC3	frameshift	c.2992_2993dupCA	p.Gln998HisfsTer9	ENST00000545968.6:c.2992_2993dupCA	ENSP00000442795:p.Gln998fs	likely pathogenic	-	likely pathogenic	PVS1, PM2	-	-	-	yes
MYBPC3	missense	c.2992C>G	p.Gln998Glu	ENST00000545968.6:c.2992C>G	ENSP00000442795.1:p.Gln998Glu	benign	-	benign	ExAC frequency	2	2,5	20	no
MYBPC3	frameshift	c.3039_3040dupCC	p.Leu1014ProfsTer7	ENST00000545968.6:c.3039_3040dupCC	ENSP00000442795:p.Leu1014fs	likely pathogenic	-	likely pathogenic	PVS1, PM2	-	-	-	yes
MYBPC3	missense	c.3065G>C	p.Arg1022Pro	ENST00000545968.6:c.3065G>C	ENSP00000442795:p.Arg1022Pro	VUS	-	VUS	PM2, PP2, PP3	5	3,6	8	no
MYBPC3	missense	c.3097C>T	p.Arg1033Trp	ENST00000545968.6:c.3097C>T	ENSP00000442795.1:p.Arg1033Trp	VUS	-	VUS	PM2, PP2, PP3	3	3	6	no
MYBPC3	missense	c.3098G>A	p.Arg1033Gln	ENST00000545968.6:c.3098G>A	ENSP00000442795.1:p.Arg1033Gln	VUS	-	VUS	PM2, PP3	3	3	5	no
MYBPC3	frameshift	c.3166dupG	p.Ala1056GlyfsTer9	ENST00000545968.6:c.3166dup	ENSP00000442795.1:p.Ala1056GlyfsTer9	pathogenic	PS4	likely pathogenic	PVS1, PM2	5	5	2	no
MYBPC3	splice donor variant	c.3190+1G>A	-	ENST00000545968.6:c.3190+1G>A	-	pathogenic	PS4	likely pathogenic	PVS1, PM2	5	5	5	no
MYBPC3	inframe indel	c.3407_3409delACT	p.Tyr1136del	ENST00000545968.6:c.3407_3409del	ENSP00000442795.1:p.Tyr1136del	likely pathogenic	PS4, PP1	VUS	PM2, PM4	5	3,8	9	no
MYBPC3	frameshift	c.3476_3477delTT	p.Phe1159TyrfsTer9	ENST00000545968.6:c.3476_3477del	ENSP00000442795.1:p.Phe1159TyrfsTer9	likely pathogenic	-	likely pathogenic	PVS1, PM2	5	5	1	no
MYBPC3	nonsense	c.3642G>A	p.Trp1214X	ENST00000545968.6:c.3642G>A	ENSP00000442795.1:p.Trp1214Ter	likely pathogenic	-	likely pathogenic	PVS1, PM2	5	4,6	7	no
MYBPC3	nonsense	c.3697C>T	p.Gln1233Ter	ENST00000545968.6:c.3697C>T	ENSP00000442795.1:p.Gln1233Ter	pathogenic	-	pathogenic	PVS1, PS4, PP1, PM2	5	4,9	12	no
MYBPC3	nonsense	c.3811C>T	p.Arg1271X	ENST00000545968.6:c.3811C>T	ENSP00000442795:p.Arg1271*	pathogenic	PS4	likely pathogenic	PVS1, PM2	5	4,9	9	no
MYBPC3	frameshift	c.431_432delGT	p.Gly144AlafsTer8	ENST00000545968.6:c.431_432del	ENSP00000442795.1:p.Gly144AlafsTer8	likely pathogenic	-	likely pathogenic	PVS1, PM2	5	5	1	no
MYBPC3	splice donor variant	c.505+1G>A	-	ENST00000545968.6:c.505+1G>A	-	likely pathogenic	-	likely pathogenic	PVS1, PM2	5	4,5	2	no
MYBPC3	missense	c.565G>A	p.Val189Ile	ENST00000545968.6:c.565G>A	ENSP00000442795.1:p.Val189Ile	benign	-	benign	ExAC frequency	2	1,6	15	no
MYBPC3	missense	c.649A>G	p.Ser217Gly	ENST00000545968.6:c.649A>G	ENSP00000442795.1:p.Ser217Gly	benign	-	benign	ExAC frequency	3	1,4	11	no
MYBPC3	splice donor variant	c.821+1G>A	-	ENST00000545968.6:c.821+1G>C	-	pathogenic	PS4, PP1	likely pathogenic	PVS1, PM2	5	5	1	no
MYBPC3	missense	c.977G>A	p.Arg326Gln	ENST00000545968.6:c.977G>A	ENSP00000442795.1:p.Arg326Gln	benign	-	benign	ExAC frequency	2	1,3	19	no
MYBPC3	frameshift	c.1293delC	p.Asp431GlufsTer19	ENST00000545968:c.1293delC	ENSP00000442795:p.Asp431fs	likely pathogenic	-	likely pathogenic	PVS1, PM2	-	-	-	yes

MYBPC3	missense	c.238G>A	p.Ala80Thr	ENST00000545968:c.238G>A	ENSP00000442795:p.Ala80Thr	VUS	-	VUS	PM2, PP2	3	3	2	no
MYBPC3	missense	c.3737T>C	p.Phe1246Ser	ENST00000545968:c.3737T>C	ENSP00000442795:p.Phe1246Ser	VUS	-	VUS	PM2, PP2	-	-	-	yes
MYBPC3	missense	c.506G>T	p.Gly169Val	ENST00000545968:c.506G>T	ENSP00000442795:p.Gly169Val	VUS	-	VUS	PM2, PP2, PP3	-	-	-	yes
MYBPC3	frameshift	c.73delA	p.Ser25AlafsTer14	ENST00000545968:c.73delA	ENSP00000442795:p.Ser25fs	likely pathogenic	-	likely pathogenic	PVS1, PM2	-	-	-	yes
MYH7													
MYH7	missense	c.1063G>A	p.Ala355Thr	ENST00000355349.4:c.1063G>A	ENSP00000347507:p.Ala355Thr	pathogenic	-	pathogenic	PS4, PM1, PM2, PP1, PP3	5	4,5	10	no
MYH7	missense	c.1208G>A	p.Arg403Gln	ENST00000355349.4:c.1208G>A	ENSP00000347507:p.Arg403Gln	pathogenic	-	pathogenic	PS3, PS4, PM1, PM2, PM5, PP1, PP3	5	5	13	no
MYH7	nonsense	c.1792A>T	p.Lys598X	ENST00000355349.4:c.1792A>G	ENSP00000347507:p.Lys598*	VUS	-	VUS	PM2	-	-	-	yes
MYH7	missense	c.1987C>T	p.Arg663Cys	ENST00000355349.4:c.1987C>T	ENSP00000347507.3:p.Arg663Cys	pathogenic	-	pathogenic	PS4, PP3, PM1, PM2, PM5	5	5	11	no
MYH7	missense	c.2146G>A	p.Gly716Arg	ENST00000355349.4:c.2146G>A	ENSP00000347507:p.Gly716Arg	pathogenic	-	pathogenic	PS4, PM1, PM2, PM6, PP1, PP3	5	4,9	10	no
MYH7	missense	c.2156G>A	p.Arg719Gln	ENST00000355349.4:c.2156G>A	ENSP00000347507.3:p.Arg719Gln	pathogenic	-	pathogenic	PS4, PP1, PM1, PM2, PM5, PP3	5	4,8	14	no
MYH7	missense	c.2207T>C	p.Ile736Thr	ENST00000355349.4:c.2207T>C	ENSP00000347507:p.Ile736Thr	pathogenic	-	pathogenic	PVS1, PM1, PM2, PP1	5	5	12	no
MYH7	missense	c.2348G>A	p.Arg783His	ENST00000355349.4:c.2348G>A	ENSP00000347507.3:p.Arg783His	likely pathogenic	-	likely pathogenic	PS4, PM1, PM2	4	4	6	no
MYH7	missense	c.2710C>T	p.Arg904Cys	ENST00000355349.4:c.2710C>T	ENSP00000347507:p.Arg904Cys	pathogenic	PS4_Moderate, PP1_Strong, PM6, PM5	VUS	PM1, PM2, PP3	5	4,5	6	no
MYH7	missense	c.2770G>A	p.Glu924Lys	ENST00000355349.4:c.2770G>A	ENSP00000347507.3:p.Glu924Lys	pathogenic	-	pathogenic	PS2, PS3, PS4, PM1, PM2, PP1, PP3	5	4,8	12	no
MYH7	missense	c.2785G>A	p.Glu929Lys	ENST00000355349.4:c.2785G>A	ENSP00000347507:p.Glu929Lys	likely pathogenic	PS4	VUS	PM1, PM2, PP3	4	4	2	no
MYH7	missense	c.3158G>A	p.Arg1053Gln	ENST00000355349.4:c.3158G>A	ENSP00000347507:p.Arg1053Gln	likely pathogenic	-	likely pathogenic	PS4, PM2, PP1, PP3	5	4,7	6	no
MYH7	missense	c.3346G>A	p.Glu1116Lys	ENST00000355349.4:c.3346G>A	ENSP00000347507:p.Glu1116Lys	VUS	-	VUS	PM2, PP2	5	4	3	no
MYH7	missense	c.3455A>C	p.Glu1152Ala	ENST00000355349.4:c.3455A>T	ENSP00000347507.3:p.Glu1152Val	VUS	-	VUS	PM2, PP2, PP3	3	3	3	no
MYH7	missense	c.4130C>T	p.Thr1377Met	ENST00000355349.4:c.4130C>T	ENSP00000347507.3:p.Thr1377Met	likely pathogenic	-	likely pathogenic	PS4, PM2, PP3	5	4,2	10	no
MYH7	missense	c.4348G>A	p.Asp1450Asn	ENST00000355349.4:c.4348G>A	ENSP00000347507.3:p.Asp1450Asn	VUS	-	VUS	PM2, PP3	3	3	5	no
MYH7	missense	c.4472C>G	p.Ser1491Cys	ENST00000355349.4:c.4472C>G	ENSP00000347507:p.Ser1491Cys	benign	-	benign	ExAC frequency	2	1,2	20	no
MYH7	missense	c.505A>G	p.Arg169Gly	ENST00000355349.4:c.505A>G	ENSP00000347507:p.Arg169Gly	likely pathogenic	PP1, PS4	VUS	PM2, PP2, PP3	4	4	2	no

MYH7	missense	c.5135G>A	p.Arg1712Gln	ENST00000355349.4:c.5135G>A	ENSP00000347507:p.Arg1712Gln	likely pathogenic	-	likely pathogenic	PS4, PM1, PM2, PP1, PP3	5	4	18	no
MYH7	missense	c.596C>T	p.Ala199Val	ENST00000355349.4:c.596C>T	ENSP00000347507:p.Ala199Val	likely pathogenic	PS4	VUS	PM1, PM2, PP3	5	4,3	4	no
MYH7	missense	c.715G>A	p.Asp239Asn	ENST00000355349.4:c.715G>A	ENSP00000347507:p.Asp239Asn	pathogenic	-	pathogenic	PS4, PM1, PM2, PP1	5	4,5	10	no
MYH7	missense	c.1988G>A	p.Arg663His	ENST00000355349:c.1988G>A	ENSP00000347507:p.Arg663His	pathogenic	-	pathogenic	PS4, PP1, PM2, PM1	5	4,9	28	no
MYH7	missense	c.2092G>A	p.Val698Met	ENST00000355349:c.2092G>A	ENSP00000347507:p.Val698Met	VUS	-	VUS	PM1, PM2, PP3	-	-	-	yes
MYH7	missense	c.2631G>T	p.Met877Ile	ENST00000355349:c.2631G>T	ENSP00000347507:p.Met877Ile	VUS	-	VUS	PM1, PM2	-	-	-	yes
MYH7	missense	c.3116A>G	p.Glu1039Gly	ENST00000355349:c.3116A>G	ENSP00000347507:p.Glu1039Gly	VUS	-	VUS	PP2, PP3	3	3	3	no
MYH7	missense	c.440G>T	p.Arg147Met	ENST00000355349:c.440G>T	ENSP00000347507:p.Arg147Met	VUS	-	VUS	PM2, PP2, PP3	-	-	-	yes
MYH7	missense	c.5159A>G	p.Asn1720Ser	ENST00000355349:c.5159A>G	ENSP00000347507:p.Asn1720Ser	VUS	-	VUS	PM2, PP3	-	-	-	yes
MYH7	missense	c.535G>C	p.Glu179Gln	ENST00000355349:c.535G>C	ENSP00000347507:p.Glu179Gln	VUS	-	VUS	PM2, PP2, PP3	-	-	-	yes
MYH7	missense	c.5615A>G	p.Gln1872Arg	ENST00000355349:c.5615A>G	ENSP00000347507:p.Gln1872Arg	VUS	-	VUS	PM2	-	-	-	yes
MYH7	missense	c.950A>G	p.Glu317Gly	ENST00000355349:c.950A>G	ENSP00000347507:p.Glu317Gly	VUS	-	VUS	PM1, PM2	-	-	-	yes
MYL2													
MYL2	missense	c.374C>T	p.Thr125Met	ENST00000228841.15:c.374C>T	ENSP00000228841:p.Thr125Met	VUS	-	VUS	PM2, PP2	3	3	6	no
MYL2	missense	c.401A>C	p.Glu134Ala	ENST00000228841.15:c.401A>C	ENSP00000228841.8:p.Glu134Ala	VUS	-	VUS	PP2, PP3	5	3,5	14	no
MYL2	missense	c.80A>G	p.Gln27Arg	ENST00000228841.15:c.80A>G	ENSP00000228841.8:p.Gln27Arg	VUS	-	VUS	PM2, PP2	4	4	2	no
MYL2	nonsense	c.190G>T	p.Glu64X	ENST00000228841:c.190G>T	ENSP00000228841:p.Glu64*	VUS	-	VUS	PM2	-	-	-	yes
MYL3													
MYL3	missense	c.170C>G	p.Ala57Gly	ENST00000292327.6:c.170C>G	ENSP00000292327:p.Ala57Gly	VUS	-	VUS	BS1, PP2	5	3,7	8	no
MYL3	missense	c.461G>A	p.Arg154His	ENST00000292327.6:c.461G>A	ENSP00000292327.4:p.Arg154His	VUS	-	VUS	PM2, PP2, PP3	5	3	8	no
MYL3	missense	c.530A>G	p.Glu177Gly	ENST00000292327.6:c.530A>G	ENSP00000292327.4:p.Glu177Gly	VUS	-	VUS	PP2, PP3	3	3	13	no
MYL3	missense	c.518T>C	p.Met173Thr	ENST00000292327:c.518T>C	ENSP00000292327:p.Met173Thr	VUS	-	VUS	PM2, PP2	3	3	2	no

PRKAG2													
PRKAG2	missense	c.245A>C	p.Gln82Pro	ENST00000287878:c.245A>C	ENSP00000287878:p.Gln82Pro	VUS	-	VUS	PM2	3	3	1	no
PRKAG2	missense	c.298G>A	p.Gly100Ser	ENST00000287878:c.298G>A	ENSP00000287878:p.Gly100Ser	benign	-	benign	ExAC frequency	3	1,46	13	no
PRKAG2	missense	c.698C>G	p.Ala233Gly	ENST00000287878:c.698C>G	ENSP00000287878:p.Ala233Gly	VUS	-	VUS	BS1	3	2,72	11	no
PRKAG2	missense	c.425C>T	p.Thr142Ile	ENST00000488258:c.425C>T	ENSP00000287878:p.Thr142Ile	VUS	-	VUS	BS1	3	3	11	no
PTPN11													
PTPN11	missense	c.556C>T	p.Arg186Trp	ENST00000351677:c.556C>T	ENSP00000340944.2:p.Arg186Trp	VUS	-	VUS	PM2, PP2, PP3	3	2,75	4	no
RAF1													
RAF1	missense	c.1837C>G	p.Leu633Val	ENST00000442415.7:c.1897C>G	ENSP00000401888.2:p.Leu633Val	pathogenic	PP2, PM2, PS3, PS2	VUS	PM2	5	4,8	11	no
RAF1	missense	c.770C>T	p.Ser257Leu	ENST00000442415.7:c.770C>T	ENSP00000401888.2:p.Ser257Leu	pathogenic	PP2, PM1, PM2, PS3, PM6_Strong	VUS	PM2	5	5	29	no
TNNC1													
TNNC1	missense	c.435C>A	p.Asp145Glu	ENST00000232975.8:c.435C>A	ENSP00000232975.3:p.Asp145Glu	VUS	-	VUS	PP2, BS1	5	3,42	7	no
TNNI3													
TNNI3	missense	c.485G>A	p.Arg162Gln	ENST00000344887.10:c.485G>A	ENSP00000341838:p.Arg162Gln	pathogenic	-	pathogenic	PM2, PP2, PP1, PS4	5	4,5	15	no
TNNI3	missense	c.497C>T	p.Ser166Phe	ENST00000344887.10:c.497C>T	ENSP00000341838.5:p.Ser166Phe	likely pathogenic	PS4	VUS	PM2, PP2, PP3	5	4,4	7	no
TNNI3	missense	c.557G>A	p.Arg186Gln	ENST00000344887.10:c.557G>A	ENSP00000341838.5:p.Arg186Gln	pathogenic	PS4, PP1	VUS	PM2, PP2	5	4,9	10	no
TNNI3	missense	c.248T>C	p.Leu83Pro	ENST00000344887:c.248T>C	ENSP00000341838:p.Leu83Pro	VUS	-	VUS	PM2, PP2	-	-	-	yes
TNNI3	missense	c.456T>A	p.Asp152Glu	ENST00000344887:c.456T>A	ENSP00000341838:p.Asp152Glu	VUS	-	VUS	PM2, PP2	-	-	-	yes
TNNT2													
TNNT2	missense	c.274C>T	p.Arg92Trp	ENST00000367318.10:c.274C>T	ENSP00000236918:p.Arg102Trp	pathogenic	-	pathogenic	PS4, PM2, PM5, PP1, PP2, PP3	5	5	11	no
TNNT2	missense	c.538A>T	p.Asn180Tyr	ENST00000367318:c.538A>T	ENSP00000236918:p.Asn190Tyr	VUS	-	VUS	PM2, PP2, PP1, PS4	-	-	-	yes
TNNT2	missense	c.844A>G	p.Lys282Glu	ENST00000367318:c.844A>G	ENSP00000236918:p.Lys292Glu	VUS	-	VUS	PM2, PP2	-	-	-	yes
TNNT2	missense	c.83C>T	p.Ala28Val	ENST00000367318:c.83C>T	ENSP00000236918:p.Ala38Val	VUS	-	VUS	PP2, BP4	5	2,3	16	no
TNNT2	inframe indel	c.487_489delGAG	p.Glu163del	ENST00000367318:c.487_489delGAG	ENSP00000236918:p.Glu173del	pathogenic	-	pathogenic	PS4, PM2, PM4, PP1	5	5	6	no

TNNT2	missense	c.832C>T	p.Arg278Cys	ENST00000367318:c.832C>T	ENSP00000236918:p.Arg288Cys	VUS	-	VUS	PP2	5	3,9	23	no
TPM1													
TPM1	missense	c.331C>G	p.Gln111Glu	ENST00000403994.9:c.331C>G	ENSP00000385107:p.Gln111Glu	VUS	-	VUS	PM2	-	-	-	yes
TPM1	missense	c.790A>G	p.Lys264Glu	ENST00000403994.9:c.790A>G	ENSP00000385107.4:p.Lys264Glu	VUS	-	VUS	PM2, PP2, PP3	3	3	2	no
TPM1	missense	c.842T>C	p.Met281Thr	ENST00000403994.9:c.842T>C	ENSP00000385107.4:p.Met281Thr	likely pathogenic	PS4, PP1	VUS	PM2, PP2	5	3,8	7	no
TPM1	missense	c.431A>G	p.Gln144Arg	ENST00000403994:c.431A>G	ENSP00000385107:p.Gln144Arg	VUS	-	VUS	PM2, PP2, PP3	-	-	-	yes
TPM1	missense	c.804A>C	p.Lys268Asn	ENST00000403994:c.804A>C	ENSP00000385107:p.Lys268Asn	VUS	-	VUS	PM2, PP2	-	-	-	yes
TTN													
TTN	frameshift	c.70437delG	p.Lys23480AsnfsTer30	ENST00000589042:c.70437delG	ENSP00000467141:p.Lys23480fs	likely pathogenic		likely pathogenic	PVS1, PM2	-	-	-	yes

Supplementary Table S3. Gene-level variant analysis, with the number (n) and the percentage (%) of variants identified in different genes [percentage is given as per class of variants (i.e. per P/LP, per VUS, per B/LB variants)]. Definitive: genes with definitive evidence for HCM association; moderate: genes with moderate evidence for HCM association; P/LP: pathogenic/likely pathogenic variant; VUS: variant of unknown significance; B/LB: benign/likely benign variant; LVH: left ventricular hypertrophy.

		P/LP, n (%)						VUS, n (%)				B/LB, n (%)		Total n (%)
		missense	nonsense	splice variant	frameshift	inframe del	novel	missense	nonsense	inframe del	novel	missense	intronic variant	
Definitive	***MYBPC3***	3 (14)	5 (23)	3 (14)	10 (45)	1 (5)	4 (18)	9 (100)	-	-	2 (22)	6 (100)	-	37 (100)
	MYH7	17 (100)	-	-	-	-	-	10 (83)	1 (8)	1 (8)	8 (67)	1 (100)	-	30 (100)
	TNNT2	1 (50)	-	-	-	1 (50)	-	4 (100)	-	-	2 (50)	-	-	6 (100)
	TNNI3	3 (100)	-	-	-	-	-	2 (100)	-	-	2 (100)	-	-	5 (100)
	TPM1	1 (100)	-	-	-	-	-	4 (100)	-	-	3 (75)	-	-	5 (100)
	ACTC1	1 (100)	-	-	-	-	-	2 (100)	-	-	1 (50)	-	-	3 (100)
	MYL2	-	-	-	-	-	-	3 (75)	1 (25)	-	1 (25)	-	-	4 (100)
	MYL3	-	-	-	-	-	-	4 (100)	-	-	-	-	-	4 (100)
Moderate	***CSRP3***	-	-	-	-	-	-	3 (100)	-	-	-	1 (100)	-	4 (100)
	TNNC1	-	-	-	-	-	-	1 (100)	-	-	-	-	-	1 (100)
	JPH2	-	-	-	-	-	-	4 (100)	-	-	4 (100)	2 (100)	-	6 (100)
Syndromic genes with isolated LVH	***ACTN2***	-	-	-	-	-	-	2 (100)	-	-	-	2 (100)	-	4 (100)
	DES	1 (100)	-	-	-	-	-	5 (100)	-	-	2 (40)	1 (100)	-	7 (100)
	FHL1	-	1 (100)	-	-	-	1 (100)	1 (100)	-	-	-	-	-	2 (100)
	RAF1	2 (100)	-	-	-	-	-	-	-	-	-	-	-	2 (100)
	PRKAG2	-	-	-	-	-	-	3 (100)	-	-	-	1 (100)	-	4 (100)
	LAMP2	-	-	-	-	-	-	-	-	-	-	2 (67)	1 (33)	3 (100)
	PTPN11	-	-	-	-	-	-	1 (100)	-	-	-	-	-	1 (100)
Other	**mitochondrial**	1 (100)	-	-	-	-	-	-	-	-	-	-	-	1 (100)
	TTN	-	-	-	1 (100)	-	1 (100)	-	-	-	-	-	-	1 (100)
